# Mineral Trioxide Aggregate as an Apexogenesis Agent for Complicated Crown Fractures in Young Permanent Incisor

**DOI:** 10.1155/2023/5597996

**Published:** 2023-07-03

**Authors:** Bibhav Dubey, Monika Rathore

**Affiliations:** Department of Pediatric and Preventive Dentistry, BBD College of Dental Sciences, BBD University, Lucknow, Uttar Pradesh, India

## Abstract

Traumatic dental injuries are extremely common in children, and trauma to developing permanent teeth can disrupt root maturation; vital pulp therapy is an appropriate treatment for these teeth. This case report describes a 9-year-old boy who suffered dental trauma while playing football, resulting in an enamel–dentin fracture with pulp exposure in the left central incisor with an open apex (Cvek's stage 3) and an enamel–dentin fracture in the right central incisor with an open apex (Cvek's stage 3). Apexogenesis with mineral trioxide aggregate was performed to preserve the neurovascular bundle, allowing normal radicular formation in the left central incisor. During a 2-year follow-up, the tooth showed no signs and symptoms, and radiographic examinations revealed no evidence of radiolucent lesions in the periapical region. This case study provides compelling evidence that the utilization of the described agent yields significant efficacy in treating traumatic fractures accompanied by pulp exposure.

## 1. Introduction

**Figure 1:**
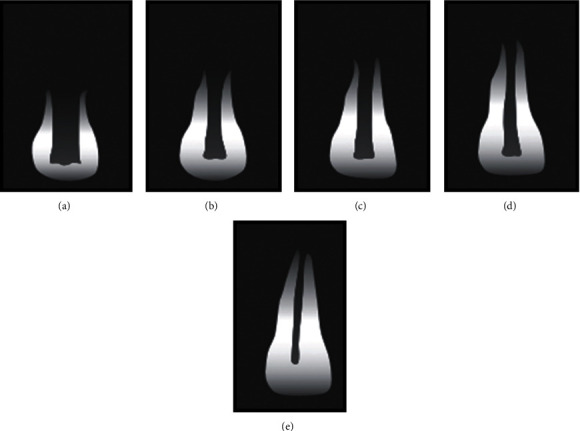
Root development stages. (a), (b), (c), (d), and (e) represent stages 1, 2, 3, 4, and 5. 1–4 = immature tooth and 5 = mature tooth. Adapted from Cvek's Figure 1 [11].

A complicated crown fracture refers to a dental injury where there is damage to both the enamel and dentin of a tooth, leading to exposure of the pulp. The prevalence of such fractures can vary between 2% and 13% of all dental injuries [1]. Typically, these injuries occur in newly erupted or young permanent teeth with immature roots [2]. Trauma to teeth with vital pulps and open apices can result in pulpal and periapical diseases. Preserving pulp vitality while allowing for root formation and apical closure is a significant challenge in these cases. Treatment planning is influenced by factors, such as the extent of pulp exposure to the oral environment, the stage of root development (Cvek's stage; Figure 1), and the time between the injury and examination [3].

When dealing with traumatized immature teeth with open apices and pulp exposure, vital pulp therapy is the preferred treatment option. Apexogenesis and other vital pulp therapies are gaining popularity due to several advantages, including shorter appointments and less technique sensitivity. Apexogenesis encourages natural root development, leading to apical closure and strengthening of the root structure [4]. In this method, the coronal portion of the pulp is partially or completely removed, usually up to the canal orifices. The remaining pulp is then capped with a suitable medicament to stimulate hard tissue formation and create a potential seal [5].

For many years, calcium hydroxide has been the primary pulp-capping agent. However, it has certain disadvantages, such as the formation of defects in the dentin bridge beneath the calcium hydroxide layer and increasing the risk of failure [6, 7]. In recent years, regenerative endodontic materials have gained popularity. Mineral trioxide aggregate (MTA) has emerged as an effective capping agent for pulp tissue healing [1, 8]. It possesses excellent sealing ability, biocompatibility, low cytotoxicity, and the capacity to induce odontoblast-like cells, which contribute to the formation of a hard barrier [9, 10]. These cells originate from the differentiation of progenitor cells that are stimulated and accumulate at the site of MTA application [8–10].

The objective of this clinical case report is to describe a partial pulpotomy procedure using MTA in a complicated crown fracture of an immature permanent incisor tooth on the left side. Additionally, the report highlights the accelerated apical closure observed in the affected tooth with an open apex during a two-year follow-up period.

## 2. Case Presentation

**Figure 2:**
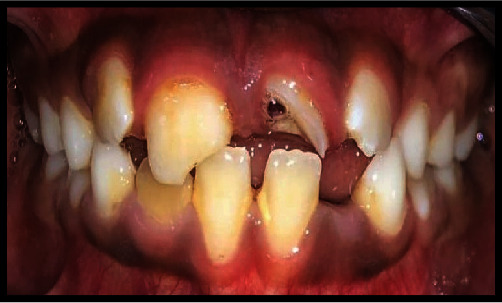
Ellis class II fracture involving tooth #11, and Ellis class III fracture involving tooth #21.

A nine-year-old boy presented to the Department of Pediatric and Preventive Dentistry with a chief complaint of dental fractures. The patient had an Ellis III fracture in the maxillary left central incisor (tooth #21) and an Ellis II fracture in the maxillary right central incisor (tooth #11) due to a traumatic injury sustained while playing football (Figure 2). The parents promptly brought the child to the department, approximately six hours after the incident occurred. The patient did not experience any spontaneous pain at the time of presentation.

**Figure 3:**
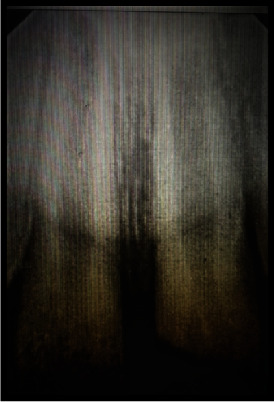
Pre-operative radiographs of teeth #11 and #21 with Cvek's stage 3.

During the intraoral examination, pulp exposure was observed in the maxillary left central incisor (tooth #21) with mild pain upon percussion. No periodontal pockets were detected, and the affected teeth showed class I mobility. Pulp vitality testing revealed a positive response to cold stimulation. The extent of pulp exposure in tooth #21 was measured to be approximately 2–3 mm. The radiographic examination did not reveal any root fractures or periradicular radiolucency in the regions of teeth #11 and #21, but it did confirm an enamel–dentin fracture in the maxillary right central incisor (tooth #11; Figure 3). According to Cvek's classification, both incisors were determined to be at stage 3 of root development; with two-thirds of root length present (Figures 1 and 3).

**Figure 4:**
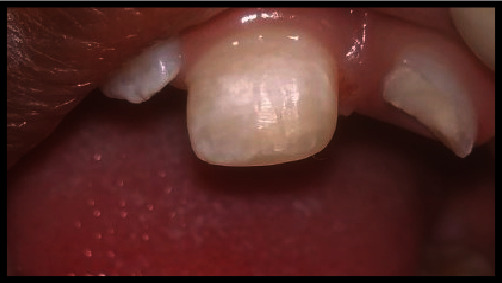
Oral prophylaxis is followed by composite resin buildup on tooth #11, with an arrow indicating MTA with Glass ionomer cement restoration on tooth #21.

**Figure 5:**
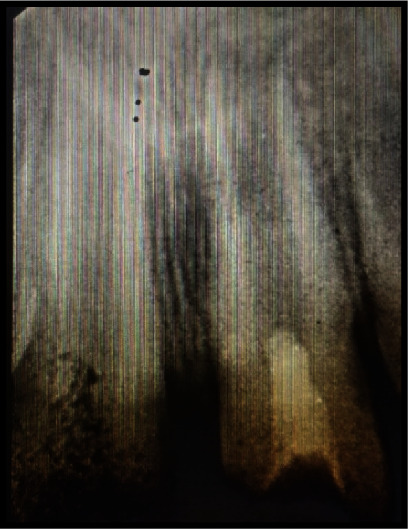
Post-operative immediate radiograph showing MTA on tooth #21.

**Figure 6:**
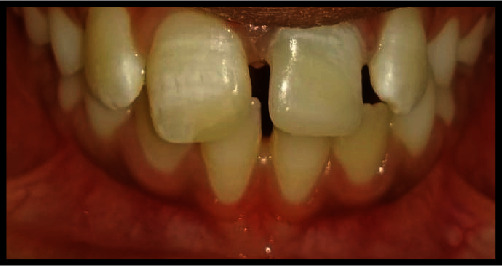
After 2 weeks of follow-up, asymptomatic and composite resin builds up on tooth #21.

**Figure 7:**
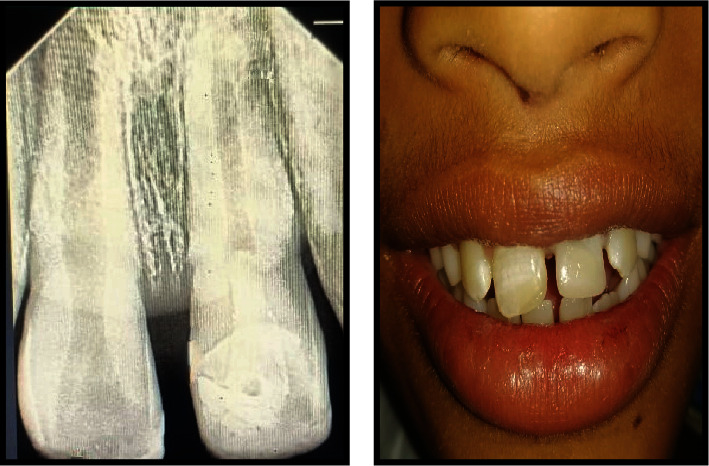
Six-month follow-up radiograph showing thickening of wall and lengthening of root length with faster apical closure on tooth #21 than tooth #11. Cvek's stage 4.

**Figure 8:**
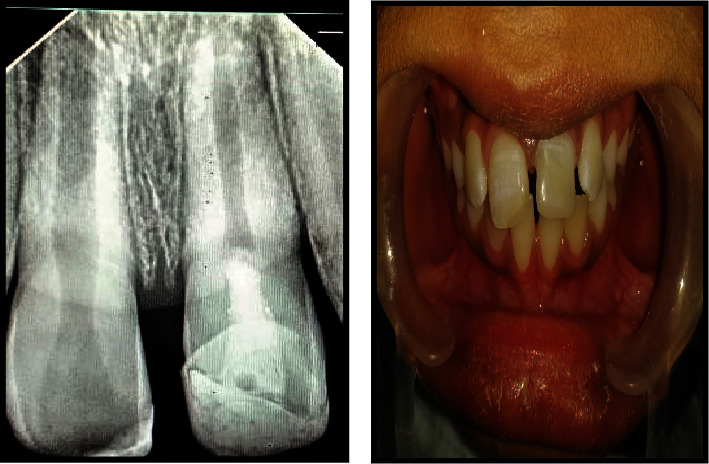
Radiograph demonstrating the faster progression of apical closure on tooth #21 with MTA than tooth #11 after 2 years. The patient had no symptoms. Cvek's stage 5.

Oral prophylaxis was performed, and the sandwich technique followed by composite buildup was carried out for tooth #11 (Figure 4). For tooth #21, the treatment plan involved a partial pulpotomy procedure, which was explained to the patient and parents. Local anesthesia (LA) was administered through the infiltration of 2% lidocaine HCl with 1 : 100,000 epinephrine (LA). Using a sterile high-speed diamond bur and water irrigation to prevent thermal damage, approximately 2–3 mm of visibly inflamed pulp and adjacent dentin were removed from tooth #21. The access cavity was then rinsed with normal saline, and the coronal pulp tissue was removed until adequate hemostasis was achieved. A moistened sterile cotton pellet was placed over the remaining pulp for 5 minutes. White MTA powder (ProRoot MTA, DENTSPLY, Tulsa, OK, USA) mixed with distilled water was applied to the exposed pulp without pressure. A moistened cotton pellet was gently placed over the MTA to facilitate its setting. After 10 minutes, the MTA was covered with glass ionomer restorative cement (GC Gold Label II, GC Fuji II Tokyo, Japan), and the patient was discharged (Figures 4 and 5). During the two-week follow-up, the patient remained asymptomatic, with no pain, periodontal pockets, mobility, or sensitivity upon cold testing. The glass ionomer restoration was partially replaced with a direct bonded composite restoration (Figure 6). Clinical and radiographic evaluations were performed at one month, three months, six months, and one-year post-treatment, with no symptoms observed. Radiographs showed increased root lengths, accelerated apical closure, complete root growth, increased thickness of the root wall, and the formation of a calcified bridge above the vital pulp (Figure 7). The periodontal ligament space appeared normal in thickness, and the continuity of the lamina dura was observed. No radiolucent lesions were detected during the six-month follow-up or in subsequent examinations conducted over one to two years (Figure 8).

## 3. Discussion

Dental health professionals should be guided when making decisions and providing the best possible care to their patients. After reviewing several dental articles on traumatic tooth injury, the International Association of Dental Traumatology and the American Academy of Pediatric Dentistry published a guideline that recommended partial pulpotomy in this clinical scenario [12, 13]. This recommendation is based on the idea that clinicians should make every effort to preserve pulp vitality in developing teeth to maintain physiologic root development and strengthen tooth resistance [12, 13].

According to research, neither the duration of the injury nor the size of the pulpal exposure (<4 mm) affects the outcome of partial pulpotomy with calcium hydroxide dressing [13, 14]. The patient in this case is young, with immature roots and open apices, which would lead to a better prognosis. The patient's age can influence the outcome of pulp treatments, as pulp in older patients tends to be more fibrotic and less capable of recovering [15–17].

Since many years ago, vital pulpotomy has used calcium hydroxide to induce coagulation necrosis, a low-grade irritation that causes undifferentiated pulp cells to undergo differentiation. These cells produce predentine, which is then mineralized, whereas the coagulated tissue is calcified [18, 19]. MTA has been suggested as the material of choice for use in vital partial pulpotomy treatment, similar to that of calcium hydroxide because it produces significantly more dentinal bridging in a shorter period of time with significantly less inflammation, and also provides a hard setting, non-resorbable surface without the presence of tunnels in the dentin barrier [19–21]. Furthermore, in the current clinical context, the partial pulpotomy with MTA performed on tooth #21 resulted in faster apical closure, thickening, and root strengthing than tooth #11. MTA is more efficient at inducing reparative dentinogenesis. One of the reasons for this is that MTA acts as a “calcium hydroxide-releasing material.” It is known to stimulate the natural wound-healing process of exposed pulps, which can result in reparative dentin formation [22, 23]. In addition to the calcium hydroxide release, in vitro studies have shown that MTA has dentinogenic mechanisms specific to itself [9, 23]. MTA can stimulate cells responsible for hard tissue formation, promoting the deposition of matrix, and mineralization. MTA also possesses several beneficial physical properties over calcium hydroxide. It exhibits good sealing ability, meaning it can effectively seal the exposed pulp from the oral environment. MTA has a lower degree of dissolution, meaning it does not degrade or dissolve as easily as calcium hydroxide. This higher structural stability ensures that MTA can provide a longer-lasting effect. Another noteworthy property of MTA is its ability to interact with phosphate-containing fluids, leading to the spontaneous formation of apatite precipitates [9, 22]. This not only explains its biocompatibility and bioactivity but also contributes to its sealing ability [22]. The formation of apatite precipitates helps create a local environment that supports the inherent wound-healing capacity of the pulp. Overall, the capacity of MTA to induce hard tissue repair in exposed pulps is influenced by its ability to maintain a conducive environment for the natural wound-healing process while providing the necessary stimulation for dentin formation. The unique properties of MTA, including calcium hydroxide release, dentinogenic mechanisms, sealing ability, structural stability, and apatite formation contribute to its effectiveness in promoting reparative dentinogenesis [22]. One of the most frequently mentioned disadvantages of MTA is discoloration. Furthermore, it appears that the primary cause of discoloration is the penetration of blood constituents into porosities within MTA, rather than the type of MTA (grey or white) [23–26]. MTA powder ingredients, such as ferric oxide, bismuth oxide, and magnesium oxide, may also be responsible for tooth discoloration [25, 26]. In the present case, a complicated crown fracture treated with MTA partial pulpotomy demonstrated successful clinical and radiographic outcomes due to its superior physical and biological properties and short follow-up period (Figure 8). This outcome can be attributed to the outstanding sealing ability of MTA, which effectively prevents the microleakage of bacteria. In a study conducted by Kararia et al. [27], a comparison was made between the sealing ability of MTA and retroplast. The researchers concluded that MTA demonstrated superior performance when compared with retroplast.

## 4. Conclusion

MTA has been scientifically proven effective in various endodontic procedures. In this study, MTA apexogenesis treatment was completed in 24 months, resulting in a calcified barrier and no need for further treatments. This suggests MTA's suitability as a pulp-capping material. However, it is crucial to note that this conclusion is based on one study, and longer clinical studies are recommended for better long-term effectiveness and safety data. Healthcare professionals should consider patient factors and case-specific requirements, and consult current research and guidelines for treatment decisions.

## Data Availability

Data supporting this research article are available from the corresponding author or first author upon reasonable request.
